# The importance of implementing inpatient virtual coverage in an endocrinology practice: lessons learned thus far from the COVID-19 pandemic

**DOI:** 10.1186/s40842-021-00118-7

**Published:** 2021-02-09

**Authors:** Marcio L. Griebeler, Kevin M. Pantalone, Ron Gambino, David Shewmon, Jay Morrow, Daniel Mendlovic, Vinni Makin, Marwan Hamaty, Sana Hasan, M. Cecilia Lansang, Keren Zhou, Bartolome Burguera

**Affiliations:** 1 Cleveland Clinic Lerner College of Medicine of Medicine of Case Western Reserve University, Cleveland, USA; 2 Endocrinology and Metabolism Institute, Cleveland Clinic, Cleveland, Ohio USA

**Keywords:** COVID-19, telemedicine, endocrinology, inpatient

## Abstract

The COVID-19 pandemic has rapidly changed the landscape of medical care and the healthcare system needs to quickly adapt in order to continue providing optimal medical care to hospitalized patients in an efficient, effective, and safe manner. Endocrinology diseases are commonly present in patients with COVID-19 and often are major risk factors for development of severe disease. The use of electronic consultation and telemedicine have already been well-established in the outpatient setting but yet not commonly implemented in the inpatient arena. This type of remote medical care has the potential to provide a reliable delivery of endocrine care while protecting providers and patients from spreading infection. This short review intends to provide the initial steps for the development of an inpatient telemedicine endocrine service to patients with endocrine diseases. Telehealth will become part of our daily practices and has a potential to provide a safe and efficient method of consultative service.

## Background

The emergency of the COVID-19 pandemic presented an unprecedented challenge to the medical community and health care system, including our own. We are observing abrupt changes that require very quick adaptations in order to allow us to continue providing optimal medical care to our hospitalized patients. During these difficult times, the implementation of telehealth became a key component of our medical practice, in both the outpatient and inpatient settings. [1]The Endocrinology and Metabolism Institute (EMI) at Cleveland Clinic quickly adapted so that we could continue providing optimal endocrinology-related care to hospitalized patients, while limiting unnecessary contact between consultants and exposed patients. This serves the purpose of curtailing the use of personal protective equipment (PPE), and minimizing the potential spread of infection to other patients hospitalized with non-COVID-19 related illnesses.

Cleveland Clinic is one of the largest health care systems in the United States, and our Institute provides inpatient coverage to multiple hospitals in the northeast Ohio region. Our Institute includes 40 endocrinologists, 22 advance practice providers and 6 endocrine surgeons and most of our team provides inpatient coverage. We see an average of more than 3000 hospitalized patients on an annual basis. Most of our inpatient care is for patients with diabetes and hyperglycemia but also thyroid, pituitary, calcium and adrenal/glucocorticoid related disorders.

## Telemedicine in the hospital setting

The use of electronic consultation (peer-to-peer e-consult) and virtual visits have already been well- established in the outpatient setting, and similar benefits/outcomes have been demonstrated when virtual visits were compared to face-to-face visits [2, 3], but they have not been used as frequently in hospitalized patients. New protocols for inpatient e-consults and remote monitoring have been implemented and it has been demonstrated that e-consults are fast and as effective as face-to- face visits [4–6]. Until recently, almost all of our inpatient endocrinology consults at Cleveland Clinic utilized the “old-fashion” face–to-face consultation, in addition to the undocumented “curbside” consultations amongst health care providers. There was a need to quickly develop an efficient and reliable e-consult option to provide hospital endocrine care in an efficient, effective, and safe manner.

Cleveland Clinic has become an Ohio healthcare leader in preparation and implementation of innovative health care delivery during the COVID 19 crisis. In addition to converting more than 90% of our outpatient encounters to virtual visits, our institute has also been a key driver and creator of new strategies to facilitate a quick conversion of face-to-face encounters to virtual visits to provide care to hospitalized patients through the following steps:

1. Cleveland Clinic created an inpatient task force to facilitate rapid implementation of inpatient virtual care during the COVID-19 pandemic accessing the appropriate work-flow processes, technology, as well as documentation and billing requirements, while still providing optimal medical care.
2. Through frequent meetings, constant stakeholder feedback, and changes in government policy, Cleveland Clinic developed an inpatient distance health playbook. EMI was one of the first institutes to pilot inpatient virtual care across multiple Cleveland Clinic hospitals.
3. The workflow stream for inpatient virtual care remains very similar to the standard face-to-face encounters. The consultant team receives the initial consult and makes the determination if direct contact with the patient is required. If no direct contact with the patient is required, the consultant team then determines if direct provider-to-provider communication is required with the primary service.
4. If direct contact with the primary service is not felt to be required, the consultant then proceeds with an asynchronous consult, or “e-consult”, which is comprised of a consultative chart review and provider-to-provider communications via documentation within a shared electronic health record. For billing purposes, the asynchronous consult requires a minimum of 5 minutes of consultative time. There is no need for verbal interaction, and the consultation can be accomplished with only documentation of a written report in the electronic health record.
5. If direct contact with the primary team is required, the consultant then proceeds with a synchronous consult. This is similar to an asynchronous consult; however, it includes both a direct verbal communication and written report (i.e., via a shared electronic health record) to the patient’s attending physician/primary service. Time provided for this type of service may be 5–10, 11–20, 21–30 or > 31 minutes of medical consultative discussion and review.
6. If a determination is made that direct contact with the patient is required, the consultant then decides if a video element is also a necessary component of the encounter. If a video element is not felt to be necessary, then the consultant would proceed with a telephone only consultation that may include 5–10, 11–20 or 21–30 minutes of medical discussion. It was noticed that very few patients required a direct face to face consult. Examples included a complex patient with diabetes on insulin pump or an unusual insulin regimen like U-500. Even patients admitted with diabetes ketoacidosis were able to be managed remotely with frequent communication with the primary team and nursing staff.
7. When a video interaction is also necessary, the consultant proceeds with a synchronous visit with the patient where there is patient contact that includes both audio and video elements. This encounter may be facilitated by a telepresenter (ancillary staff at bedside), but this not required. This encounter can be billed at three different levels according to either time alone, or by complexity: ≥30 min or low complexity decision making, ≥ 50 min or moderate complexity decision making or ≥ 70 min or high complexity decision making
8. As of the present time, subsequent consultative inpatient medical care is also being provided via telehealth encounters, and we are also exploring the possibility of providing follow-up care via asynchronous visits.
9. Cleveland Clinic is still in the early stages of optimization of the work flow for inpatient telehealth consultative visits. If patient interaction is required, the consultant will contact the hospital unit staff who calls into the patient room to determine if the patient can communicate independently via mobile device. If a personal phone is available the unit staff asks the patient to logon to their app (facetime/google due/Doximity) to prepare for the consultant call. If a personal phone is not available, the consultant and patient are connected through communal iPad or inTouchHealth Device where available. After the visit is finalized, the nurse helps the patient log out of the device, cleans it, and returns to the central station. If neither of the above interactions are possible, the consultant will reassess the situation and determine if a face-to-face encounter is now most appropriate, or proceed with contacting with the patient directly via their room-assigned land line telephone for an audio element-only encounter. Sometimes the consultation may not meet billing criteria; during these circumstances a plan of care will be formulated with clear recommendations to the primary team and nursing staff.
10. Our inpatient health care task force developed new consult and progress note templates in our EHR (EPIC MyPractice®) that meet all required elements for billing and documentation. These templates can be found through smart phrases. Billing can be based on the visit components and their complexity, or actual time spent. Billing codes can be visualized in Table 1. The task force also developed numerous educational documents including a telehealth resources section where the playbook is easily accessible to providers.

**Table 1:** For telemedicine services with a house officer, the teaching physician must be physically present with the HO who is providing the service, or present through interactive telecommunications technology for the key portions of the service allowing the teaching physician to furnish assistance and direction. The house officer or teaching physician can add documentation: “*Dr X was present for the key portions of the E/M service provided and agreed with the management.”*

Reporting Non-Face to Face Inpatient Care During Pandemic		
**Document the type of connection used (video or telephone) and if telephone only, include that video was not available. If the provider is not in the facility when providing the service, add GT modifier to CPT code**		
**Non-Face to Face Inpatient Care**	**CPT Codes**	**Comments**
Consult to Inpatient Team Asynchronous	99451	Written report provided in electronic chart to the requesting physician/qualified professional 5–29 min of medical consultative time No patient contact
Consult to Inpatient Team Synchronous	99446–5–10 min 99447–11–20 min 99448–21–30 min 99449 - > 31 min	Do not report more than once in 7-day period. Do not report if consultant saw patient in last 14 days. Only physicians may report. Do not add GT Modifier to these codes No patient contact
Consult to patient Synchronous Telephone only	99441–5–10 min 99442–11–20 min 99443–21–30 min	Patient contact but audio only Must not originate from a related E/M service provided within the previous 7 days Must not lead to an E/M service or procedure within the next 24 hours
Consult to patient (initial visit) Synchronous Virtual Visit (video call)	99251 – level one 99252 – level two 99253 – level three 99254 – level four 99255 – Level five	Patient contact with audio and video These codes may be reported by MD, APP, or HO if Teaching Physician participates in key portion of service and only teaching physician time can be counted.
Subsequent Inpatient Visit Synchronous Virtual Visit	99231 – level 1 99232 – level 2 99233 – level 3	Patient contact with audio and video Use same coding criteria as in-person visits. These codes may be reported by MD, APP, HO if Teaching Physician participates in key portion of service
11. In an effort to cut down PPE use and nurse contact with patients we discussed the use of continuous glucose monitoring (CGM). At the present time our experience is limited and the nursing staff felt that checking fingersticks (standard medical care) caused minimal disruption of their workflow as they still have to enter COVID-19 patients’ room.
12. The Endocrinology and Metabolism Institute had an average of 2000 monthly encounter visits prior to COVID pandemic. During the peak of the pandemic the average visits went down to around 1000 monthly encounters. In one of the pilot hospitals the daily census went down from 30 patients to around 15 patients with less than 5% of face to face visits, 30% asynchronous visits, 40% synchronous e-consults and the remaining virtual face to face visits or not billable due to technical issues.

## Conclusion

The COVID-19 pandemic has changed the landscape of medical care, including in the inpatient setting. Telehealth will become part of our daily practices and it has the potential to provide a safe and efficient means of providing consultative service, while also protecting patients and providers during this difficult time. Moving forward, we plan to evaluate outcomes and the quality of care provided via inpatient telehealth services and hypothesize that they will be similar to that obtained via with the standard face-to-face encounters. We hope this information provided will assist the endocrinology community all over the country-and all over the world- implement and provide efficient, safe, and high-quality medical care to hospitalized patients.

## Data Availability

Not applicable

## Abbreviations

EMI: Endocrinology and Metabolism Institute
